# Zinc nanoparticles mitigate azoxystrobin and its nanoencapsulation-induced hepatic and renal toxicity in rats

**DOI:** 10.1080/13510002.2025.2491318

**Published:** 2025-04-20

**Authors:** Nashwa Elshaer, Ahmed M. Eldeeb, Ahmed A.A. Aioub, Ahmed S. Hashem, Soumya Ghosh, Lamya Ahmed Alkeridis, Mohammed Ali Alshehri, Mustafa Shukry, Daklallah A. Almalki, Hind A. Alkhatabi, Mohamed Afifi, Ammar AL-Farga, Mohamed A. Hendawy, Ahmed E.A. El-Sobki

**Affiliations:** a Plant Protection Department, Faculty of Agriculture, Zagazig University, Zagazig, Egypt; b Stored Product Pests Research Department, Plant Protection Research Institute, Agricultural Research Center, Kafr El-Sheikh, Egypt; c Natural & Medical Science Research Center, University of Nizwa, Nizwa, Oman; d Department of Biology, College of Science, Princess Nourah Bint Abdulrahman University, Riyadh, Saudi Arabia; e Department of Biology, Faculty of Science, University of Tabuk, Tabuk, Saudi Arabia; f Department of Physiology, Faculty of Veterinary Medicine, Kafrelsheikh University, Kafrelsheikh, Egypt; g Biology Department, Faculty of Science and Arts, Al-Baha University, Al-Mikhwah, Saudi Arebia; h Department of Biological Sciences, College of Science, University of Jeddah, Jeddah, Saudi Arabia; i Department of Biochemistry, Faculty of Veterinary Medicine, Zagazig University, Zagazig, Egypt

**Keywords:** Azoxystrobin, nano-Azoxystrobin, zinc nanoparticles, antioxidant enzymes, histopathological study, immunohistochemical study, biochemical parameters, renal toxicity

## Abstract

This study sought to ascertain if zinc nanoparticles (ZnNPs) could lessen the toxicity of azoxystrobin (AZ). This naturally occurring methoxyacrylate is one of the most often used fungicides in agriculture in male albino rats. Six sets of 60 mature male albino rats were randomly assigned: control (distilled water), Azoxystrobin formulation (AZOF), Azoxystrobin nano-formula (AZON), ZnNPs, AZOF + ZnNPs, and AZON + ZnNPs. Blood and tissues were obtained for further immunohistochemical, pathological, and biochemical examination. The results showed that exposure to AZOF and AZON significantly increased the levels of the oxidative stress indicators glutathione peroxidase (GPx), catalase (CAT), superoxide dismutase (SOD), and malondialdehyde (MDA). Additionally, AZOF significantly impacts liver function bioindicators, including aspartate aminotransferase (AST) and alanine aminotransferase (ALT) levels. AZOF and AZON induced damage to the liver and kidney by disrupting vascular dilatation and causing hemorrhages, apoptosis, inflammatory lymphocytes, and necrosis. Furthermore, co-administration of ZnNPs with fungicides (AZOF and AZON) can gently enhance the alterations of oxidative stress and liver function bioindicators levels. These findings showed that ZnNPs could help male rats receiving AZ treat their histologically abnormal liver and kidney.

## 1. Introduction

Even though pesticides are fundamental to crop production, their application and manufacture adversely influence the environment. The ubiquitous of these pesticides typically results in residues, offering urgent threats to all species, including humans [1]. Fungicides are used extensively due to their systemic, protective, and curative functions to prevent crop fungal infection [2, 3]. In agriculture, strobilurin fungicides are extensively used to control pathogenic infections, including white mold, leaf spots, and collar blasts [4]. However, strobilurins have been classified as low-risk fungicides, and there are considerable public health risks due to their ubiquitous and prolonged use in agriculture and manufacturing [5]. Fungicides impact ecological hazards by disturbing the soil microbial communities [6].

The synthetic fungicide Azoxystrobin (Az) is an analog of the fungus metabolites strobilurins and oudemansins. It impedes electron transport in fungal mitochondria [7]. Azoxystrobin's efficacy in crop protection is significantly constrained by its low solubility in aqueous solution [8]. Several studies showed that Az induces liver and kidney damage as demonstrated by the modification of bioindicators (serum aspartate aminotransferase (AST), alanine aminotransferase (ALT), alkaline phosphatase (ALP)). Furthermore, antioxidant enzyme activity is affected by a redox imbalance that promotes oxidative stress. Malondialdehyde (MDA) is a byproduct of lipid peroxidation from membrane damage, serving as a marker rather than an activator. It can cross-link erythrocyte phospholipids and proteins. Glutathione (GSH), a low-molecular-weight antioxidant, acts as an enzyme cofactor, radical scavenger, and substrate for oxidation reactions, supporting redox balance. Inhibition of enzymes like superoxide dismutase (SOD), catalase (CAT), and GSH disrupts this balance, weakening the cell's oxidative defenses [9, 10].

Many countries have reported detecting AZ in streams, surface water, and groundwater [11, 12]. Az abuse may adversely impact non-target species and cause quinol oxidation inhibitor resistance, reducing species diversity and hurting the ecosystem. Therefore, increasing the bioavailability of Az has significant scientific and applied research benefits. The development of pesticide formulations using nanotechnology has the potential to significantly improve the ecological environment and enhance the efficacy of pesticides [13]. Nanoparticles have excellent surface/volume ratios and better permeabilities, which can lead to superior control effects [14, 15]. Superior absorption, prolonged pest protection, and minimum application dose reduction of 50% are all characteristics of the nano-Azoxystrobin (N-AZ) [16]. A poorly soluble AZ fungicide nanosuspension formulation was developed to enhance antifungal efficacy while reducing environmental impact [17]. While encapsulating pesticides in nanoparticles offers these advantages, it also introduces potential risks, as nanomaterials may exhibit borderline cytotoxicity and genotoxicity. Higher organisms readily absorb these nanoparticles, raising concerns about cytotoxic effects on mammals. Therefore, while nanosuspensions may improve the delivery and efficacy of pesticides, they also pose significant health and environmental risks that warrant careful evaluation.

Zinc (Zn) performs a crucial metabolic role, particularly in the immune system. Moreover, Zn supplementation may be essential to avoid oxidative damage [18, 19]. Although the nanoform of a compound is not always advantageous regarding toxicological concerns, nanoforms can access more organelles within the body, where their efficacy may be boosted. Several studies have demonstrated that zinc nanoparticles (ZnNPs) are systemically absorbed, raising the Zn content in various tissues [20]. Pesticides may cause modifications in enzyme activity associated with antioxidant defense mechanisms [1]. More studies showed that the hormonal disturbances and oxidative damage caused by pesticides have been mitigated by elements like zinc, selenium, or titanium in rats [21, 22]. Recently, the interaction of ZnNps with pesticides has received a lot of interest due to its extensive use in agriculture and its ability to provide antimicrobial activity [23–25]. Consequently, ZnO nanoparticles could create biodeterioration-resistant construction materials and protective coatings [26].

By changing active substances' colloidal and surface characteristics, encapsulation offers a method for turning liquids into solids [27]. Nanomaterials' physical and chemical characteristics differ from those of macroscopic materials, so they have significant applications in fields like pharmacology and biology. Nanomaterials have unique surface characteristics, are extremely small, and exhibit quantum size effects [27]. High polymer materials with light, temperature, humidity, enzyme, and soil pH sensitivity have been used to administer pesticides and create nano-microcapsules or nanospheres. The use of nanomaterials technology in pesticides has advanced significantly in recent years. Particularly, controlled release and targeted delivery of nanoparticles can enhance the efficacy of pesticides while lowering residue and pollution. Due to their small size, pesticide nanocapsule formulations used to spray fields have increased pesticide droplet ductility, wettability, and target adsorption [28–31]. This makes these techniques more efficient and environmentally friendly.

As far as we are aware, no research has been reported concerning the role of ZnNPs against the toxicity of Az fungicide and its nanoform (AZON) in vivo system, taking into consideration that ZnNPs have no adverse effects when added to the drinking water of the animals. Therefore, the current study investigated the potential impact of these nanoparticles to relieve the toxicity of Az and AZON on the immune system and their industrial and medicinal applications to eliminate this fungicide from rats.

## 2. Materials and methods

### 2.1 Chemicals used

El-Gomhouria company (Egypt) provided the Polysorbate 80 (Tween 80), Polyethylene glycol 6000 (PEG), and ethanol. Distilled water (18 0; Sartorius-arium@611VF) was utilized in this study. Zinc powder and Azoxystrobin, 20% wp, were purchased in bulk.

### 2.2 Preparation and characterization of nanoencapsulation and ZnNPs

According to Yang et al. [32], the melt dispersion was used to create the nanoencapsulation of zinc and Azoxystrobin 20% (w/w) wp. At 65°C, several PEG 6000 components were heated. The mixture was stirred with a glass rod to ensure a uniform distribution. It was then refrigerated at 25°C, grounded, sieved (with a sieve mesh of 200), and kept in an airtight container until further testing. To prevent any significant impact on the characteristics of the nanoparticles, the procedure parameters used to prepare all the nanoencapsulations were carefully controlled. Sesame seeds were rinsed, then dried, and ground. 100 ml of distilled water was combined with 20 g of seed powder and was heated for 45 min at 75°C [33]. The extract was obtained and kept at 4°C. 20 ml of the sesame seed extract was mixed with 80 ml of a one mM ZnSO47H2O solution to prepare ZnNPs. At 60°C, the solution is sonicated for one hour. The color was yellow, indicating ZnNP formation, according to Meva, Segnou [34].

To determine the isotropy and morphology of each sample, one drop was analyzed under a polarized microscope (Olympus BX-51) linked with an LC digital camera (PL-A662) using image analyzer software (PixelLINK). Droplet size (d.nm), viscosity (cP), poly dispersivity index (PDI), zeta potential, zeta deviation, and conductivity were determined as described previously by Arancibia, Navarro-Lisboa [35]. The functional groups were identified using a Fourier transform infrared spectrophotometer (FT-IR). The spectrum was scanned through the frequency range of 4000–500 cm^−1^ to characterize the compatibility of the nanoencapsulation and its constituents (PEG).

## 2.3. Animals

Sixty mature male albino rats (*Rattus norvegicus*), weighing 120–150 g, were purchased from the unit of animal housing, Veterinary Medicine Faculty, Zagazig University, Egypt. Animals were housed in stainless steel cages with a rigorous 12:12 h light–dark cycle, with temperature at 22°C. The test animals acclimated to the lab environment for a week before the experiments. Methodology adhered to regulations established by the Committee of Research Ethics for Laboratory Animal Care at the Faculty of Veterinary Medicine at Zagazig University, Zagazig, Egypt (approval no. ZU-IACUC/2/F/92/2023).

### 2.4. Experimental design

A total of six groups of ten rats each were formed at random according to previous studies [24, 36]. As a control, the first group received oral distilled water. In contrast, the second and third groups received doses of Azoxystrobin formulation (AZOF) and Azoxystrobin nano-formula (AZON) at 0.27 and 0.27 g/kg bwt, respectively, following [9]. The fourth group received Zn nanoparticles (ZnNPs) orally at a dose of 1 ml (10 mg/kg body weight). The fifth and sixth groups administered oral treatments of AZOF + ZnNPs and AZON + ZnNPs at the doses above. The results of earlier investigations [24, 36]. For 21 days, each group received treatment three times per week and monitored daily for any hazardous side effects. Food and water were freely available to rats. The rats' body weight was calculated weekly, and any disease symptoms were noted.

The body weight gain was measured by the formula below:

$$\mathrm{Body}\;\mathrm{weight}\;\mathrm{gain}=\frac{(\mathrm{Final}\;\mathrm{body}\;\mathrm{weight}-\mathrm{Initial}\;\mathrm{body}\;\mathrm{weight})x^{2}}{\mathrm{Initial}\;\mathrm{body}\;\mathrm{weight}}\;\times 100$$

### 2.5 Preparation of samples

Rats were weighed following an overnight fast after 22 days of therapy. Diethyl ether was used to euthanize the animals, and blood and tissue samples from the control and treated groups were taken by using a tiny, sterile glass capillary to puncture the retro-orbital sinus for further analysis. In preparation for a hematological study, the samples were taken in tubes with 10% (w/w) EDTA acting as an anticoagulant. A portion of the blood samples was stored in tubes at room temperature for 30 min to coagulate. Following that, the samples were centrifuged at 3000 rpm for 20 min. The resulting serum was kept at 20°C for additional testing (measurements of liver enzymes, antioxidant enzymes, and oxidative stress). Liver and kidney tissues were removed, washed with saline, and preserved in neutral formalin 10% (w/w) to determine oxidative stress parameters such as biochemical, histopathological alterations, and immunotoxicity.

### 2.6 Determination of antioxidant status markers and liver function

Nitroblue tetrazolium reduction was used to measure the activity of superoxide dismutase (SOD) [37]. According to Paglia and Valentine [38], glutathione peroxidase (GPx) activity was determined in brief Glutathione peroxidase (GPx) activity was determined by monitoring the rate of NADPH oxidation in the presence of glutathione (GSH) and a substrate, cumene hydroperoxide, using a spectrophotometer at 340 nm. The reaction mixture contained phosphate buffer (pH 7.0), GSH, NADPH, glutathione reductase, and cumene hydroperoxide as the substrate. GPx catalyzes the reduction of cumene hydroperoxide using GSH, which is subsequently regenerated by glutathione reductase in the presence of NADPH. NADPH is oxidized to NADP + during this reaction, decreasing absorbance at 340 nm. The rate of NADPH oxidation is directly proportional to GPx activity. MDA levels, indicating the rate of lipid peroxidation in tissue samples, were measured using the method described by Ohkawa et al. [39]. Briefly, each reaction mixture included 0.1 mL of sample, 0.2 mL of 8.1% (w/w**)** sodium dodecyl sulfate (SDS), 1.5 mL of 20% acetic acid, and 1.5 mL of 0.8% (v/v) TBA solution. The pH was adjusted to 3.5, and distilled water was added to bring the volume to 4.0 mL. Then, 5.0 mL of an N-butanol and pyridine mixture (15:1, v/v) was added and shaken thoroughly. Following centrifugation at 4000 rpm for 10 min, the absorbance of the organic layer was read at 532 nm. The Lowry method determined protein concentrations in tissue samples using a Spectronic UV 120 spectrophotometer [40]. The catalase activity (CAT) was measured following the method of Aebi [41], with modifications to quantify CAT's role in H_2_O_2_ degradation specifically. Tissue samples were prepared and incubated with H_2_O_2_, and the decrease in H_2_O_2_ concentration was monitored by measuring absorbance at 240 nm, which specifically reflects H_2_O_2_ decomposition. Parallel assays were conducted in the presence of aminotriazole, a selective catalase inhibitor, to isolate the catalase-specific activity. By comparing results from aminotriazole-treated samples with untreated controls, CAT-specific activity was determined by subtracting the inhibited (aminotriazole) values from total peroxidase activity. Results were expressed in units per gram of tissue (U/g), indicating CAT-specific contribution to H_2_O_2_ breakdown. The activities of alanine aminotransferase (ALT) and aspartate aminotransferase (AST) were measured using the colorimetric method of Reitman and Frankel [42]. In this assay, the enzyme reactions are initiated by incubating the sample with a substrate solution containing alanine and α-ketoglutarate for ALT, and aspartate and α-ketoglutarate for AST. ALT and AST catalyze the transfer of amino groups, producing pyruvate and oxaloacetate, respectively. These products then react with a chromogenic reagent, 2,4-dinitrophenylhydrazine, forming a colored hydrazone complex. After a fixed incubation time, sodium hydroxide is added to stop the reaction and develop the color. The absorbance of the resulting color is measured at 505 nm, with the intensity directly proportional to the ALT or AST activity in the sample. Enzyme activities are expressed in units per liter (U/L) based on calibration with standard enzyme solutions.

### 2.7 Tissue preparation for histopathological examination

Rat kidney and liver tissue samples that had been formalin-preserved underwent automated tissue processing. A two-step initial fixation followed dehydration. During fixation, the tissue is submerged for 48 h in 10% (v/v) buffered formalin, then rinsed with distilled water for 30 min. To provoke dehydration, the tissues were then exposed to increasing concentrations of alcohol (70% (w/w), 90% (w/w), and 100% (w/w)). The tissue was first exposed to 70% (v/v) alcohol for 120 min, followed by 90% (v/v) alcohol for 90 min, and then two cycles of 100% alcohol for an hour each. The tissues were immersed in a solution of 50% (v/v) alcohol and 50% (v/v) xylene for an hour, followed by 1.5 h in pure xylene. Afterward, the samples were coated with molten paraffin wax. Paraffin slices (4–5 µm) were stained using hematoxylin and eosin [43]. The stained tissues were checked for abnormalities such as apoptosis, necrosis, degeneration, inflammation, and circulatory disturbances.

## 2.8 Tissue preparation for immunohistochemical examination

According to Eissa and Shoman [44], immunohistochemistry techniques were used. Tissue sections were frequently microwaved to differentiate the antigen epitopes [45]. The primary antibody localizes the specificity of the reaction by binding it to the relevant antigen. The secondary antibody enhances the reaction's amplification in conjunction with its associated enzyme, boosting the test's sensitivity. The Biotin–Streptavidin (BSA) method was used to visualize the markers [46]. The chromogen diaminobenzidine (DAB) was selected because it enables permanent preparation.

### 2.10 Staining procedure

Paraffin slices were immersed in Xylene for a whole night, after mounting on positive-charged glass slides (Biogenex, USA). The slices were then diluted with ethanol at concentrations of 100% (v/v), 95% (v/v), 75% (v/v), and 50% (v/v) before being placed in distilled water. Slides were inserted in an open plastic vessel with adequate citrate buffer (antigen retrieval solution, pH 6). The plastic container was placed in an open plastic tray to prevent boil-over. Slides were heated for 5 min at power 10 in a microwave oven (Samsung 800 Watts). The container's fluid level was checked to keep the slides from drying out, and water was added as needed. The container was taken out of the oven and given 15 min to cool. The slides were washed with deionized water, followed by 5 min in phosphate buffer saline (PBS). An endogenous peroxidase-blocking reagent comprising hydrogen peroxide and sodium azide was applied to tissue slices (DAKO peroxidase blocking reagent, Cat. No. S 2001). After the slides were dried, one to two drops of the primary antibody caspase-3 (Cat. No. PAI-29157, Thermo Fisher Scientific Co., USA) were added after dilution by PBS (1:1000) were applied to the sections. Slides were incubated for 60 min at room temperature; after that, slides were rinsed for five min with PBS. The Secondary Antibody (EnVision + System, Horseradish Peroxidase – HRP) DAKO (Agilent Technologies) was applied for 20 min at room temperature. The slides were then rinsed with PBS as previously. The slides were exposed to 1–2 drops of diaminobenzidine (DAB) for 10-20 min until the desired brown color was achieved and then rinsed in the buffer. Sections were submerged in distilled water before being stained with Mayer's hematoxylin (Hx) counterstain for 3-5 min. Sections were cleaned in tap water and then separated in acid-alcohol before being cleaned again in water. Slides were mounted in Canadian balsam after being allowed to dry in the air.

### 2.11 Morphometric analysis.

The camera used for analyzing the slides was an Olympus LC20-Japan fixed on an Olympus BX-50 microscope (Tokyo, Japan). Video Test Morphology 5.2 software (Russia) was used to analyze the resulting images on an Intel® Core I3® based computer. The area percentage of Caspase-3 positive expression was calculated by the system. Three slices per tissue were photographed at 200 µm apart. The existence of positive cells was determined using image analysis software (JID801D) by randomly selecting three views per slice. Automatic calculations were made to determine the positive cells' average grayscale. An average grayscale representation of immunoreactive intensity was used [47].

### 2.12 Statistical analysis

Data were presented as mean ± standard error of the mean (SEM). Statistical analysis was performed using Statistica 9.0 software. Shapiro–Wilk tests were initially conducted to assess the distribution of the data. A one-way ANOVA and Tukey's posthoc and Duncan's multiple range tests were employed to examine group differences. Statistical significance was set at *p* < 0.05.

## 3. Results

### 3.1 Characterization of Azoxystrobin nanoform and ZnNPs

Nano-azoxystrobin showed a Z-average size of 418.8 nm and conductivity of 0.036 mS/cm. Additionally, the zeta potential of AZON was slightly highly negative (−14.1 ± 3.15, mean ± SD) (Figure S1A & B), and the PDI value was somewhat higher (0.466), with a low viscosity of 0.8872 cP, which the substance's low oil content may have caused. On the other hand, ZnNPs were 232.6 nm in size and had a 0.754 PDI and conductivity and viscosity values recorded 0.019 mS/cm and 0.8872 cP, respectively. Furthermore, ZnNPs had a negative zeta potential (−16.1 ± 4.49, mean ± SD) (Figure S1C & D).

### 3.2 Effects on body weight oxidative stress biomarkers and antioxidant enzyme activity

Control animals show consistent weight gain under stable conditions, while AZOF and AZOF + ZnNPs treatments significantly reduce weight variation, potentially due to impaired metabolic efficiency or growth. ZnNPs and AZON + ZnNPs treatments show minimal or positive weight changes, suggesting protective or compensatory effects. Food and water consumption trends mirror these findings: control and ZnNP-treated groups maintain stable intake, while AZOF-treated groups have reduced intake, indicating potential stress or toxicity. Overall, AZOF appears to impact metabolic health negatively, whereas ZnNPs may offer protective benefits in combination treatments, highlighting the need for further research into these mechanisms (Figure S2). As shown in Table 1, the serum levels of antioxidant enzymes (SOD, CAT, and GPx) and oxidative stress indicators (MDA) were statistically different in treated animals compared to control animals. AZOF exposure increased GPx while causing remarkably oxidative damage, as evidenced by deficiencies in SOD, CAT, and the antioxidant MDA. Compared to rats treated with AZOF alone, concurrent treatment of AZOF + ZnNPs significantly increased SOD levels. AZON-exposed rats showed a significant reduction in SOD and MDA levels and a significant increase in GPx and CAT levels. Unexpectedly, rats administered with AZON + ZnNPs showed a substantial reduction in SOD values compared to AZON-treated rats. At the same time, the administration of ZnNPs concurrently with AZON had improved levels of GPx, CAT, and MDA compared to untreated rats, as shown in Table 1.

**Table 1.** Changes in Superoxide dismutase (SOD), Glutathione peroxidase (GPx), Catalase (CAT), and Lipid Peroxidation (MDA) activities of the Wistar albino rats, Rattus norwegicus homogenates treated with azoxystrobin.

Treatment	SOD (U/ml)	GPx (U/L)	CAT (U/L)	MDA (U/ml)
Control	224.16 ± 3.814^e^	63.80 ± 5.014^a^	532.06 ± 5.683^d^	58.3 ± 0.918^f^
(AZOF)	50.59 ± 1.613^a^	83.34 ± 3.354^b^	470.40 ± 1.906^b^	56.57 ± 1.241^e^
	(77.43%)	(−30.62%)	(11.58%)	(49.27%)
(AZON)	142.55 ± 2.608^c^	94.54 ± 3.891^c^	582.14 ± 5.252^e^	43.62 ± 0.650^c^
	(36.40%)	(−48.18%)	(−9.41%)	(25.18%)
(ZnNPs)	166.14 ± 3.274^d^	87.15 ± 2.570^bc^	469.45 ± 7.800^b^	37.41 ± 0.852^b^
	(25.88%)	(−36.59%)	(11.76%)	(35.83%)
(AZOF) + (ZnNPs)	165.92 ± 4.655^d^	85.10 ± 4.456^b^	238.94 ± 7.422^a^	32.17 ± 0.945^a^
	(25.98%)	(−33.38%)	(55.09%)	(44.81%)
(AZON) + (ZnNPs)	85.78 ± 1.627^b^	65.50 ± 4.232^a^	517.58 ± 4.555^c^	54.2 ± 0.897^d^
	(61.73%)	(−2.66%)	(2.72%)	(7.03%)

Values are shown as means ± SE; **^a^**^–f^ Mean with different subscript letters within one column are significantly different *P* ≤ 0.05. The percentage of changes in biochemical parameters was calculated according to Söğüt, Şenat [74] as Δ parameter (%) = (control value − reatment value)/(control value) × 100.

### 3.3 The impact on hepatic function biomarkers

**Figure 1.**
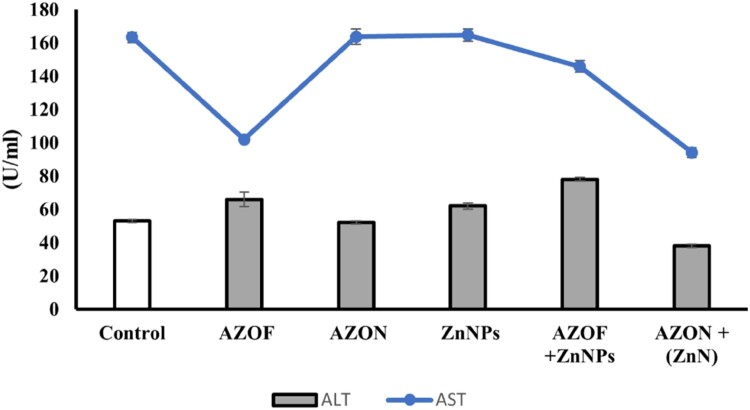
Effects of AZOF, AZON, ZnNPs, and the mixtures on liver enzymes (ALT, AST). Each measurement was done at least in triplicate samples, and data was represented as mean ± SE.

Figure 1 illustrates that rats treated with AZOF exhibited significant repression of AST levels, while rats treated independently with AZON or ZnNPs showed no significant change. However, in contrast to the pesticides used alone, the AST level markedly decreased when ZnNPs were supplied along with AZOF or AZON. In addition, ALT levels considerably increased in all treated animals, except for rats treated with AZON + ZnNPs, which had a considerable decrease.

### 3.4 Histopathological findings

**Figure 2.**
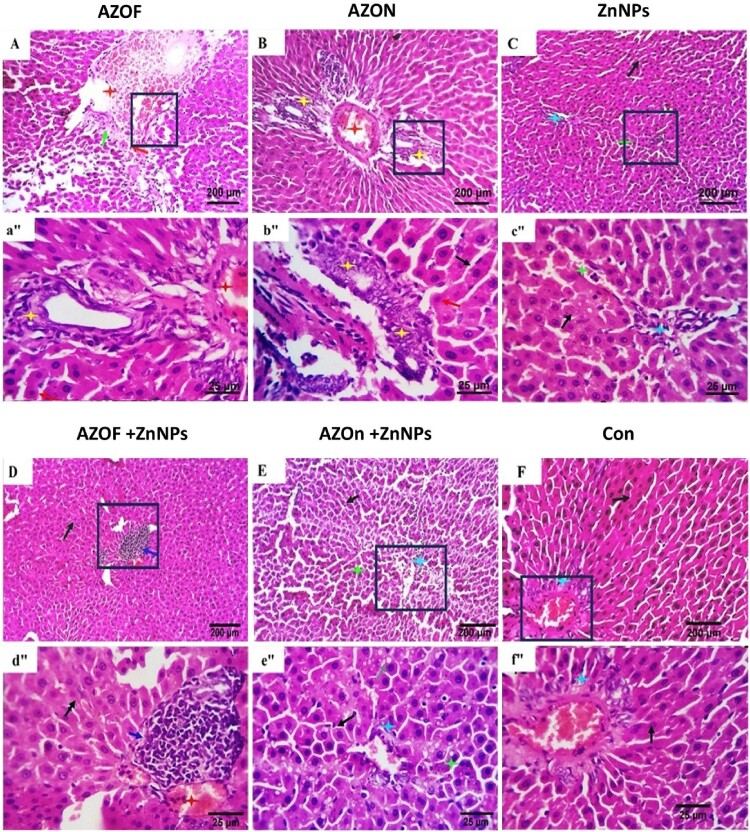
Representative photomicrographs from the livers of different experimental groups show normally arranged cords and active (F, f), occasionally binucleated hepatocytes (black arrows). The sinusoids comprise active phagocytic hypertrophic Von-Kupffer cells (green asterisks). The portal triad structures appear in an excellent histologic configuration (light blue asterisks). AZOF-treated rats (A, a″): demonstrate marked vascular dilatation with occasional interstitial hemorrhages (), cellular disorganization (black asterisks), and apoptosis of a variable number of cells (red arrows), focal degenerative and necrotic changes (green arrow) beside proliferative biliary reaction (yellow asterisks). Hepatoportal biliary proliferation (yellow asterisks), round cells (lymphocytes and plasma cells) infiltration (white asterisk), and scattered hepatocellular degenerative and apoptotic changes (red arrow) are seen in AZON-treated rats (B, b″). Mild portal and interstitial round cells infiltration (light blue asterisks) with apparently normal hepatocellular histo-morphological structures (black arrows) are apparent in ZnNPs (C, c″) and AZOF + ZnNPs-treated rats (D, d″). The same changes can be seen in AZON + ZnNPs treated rats (E, e″) with a moderate reactive portal round cell infiltration (dark blue arrow). (H&E stain). Scale bar in (A–F): 200 µm, (a″–f″): 25 µm.

Hepatic tissue of untreated rats, which exhibited regularly spaced cords and occasionally binucleated hepatocytes, was cut into serial slices for examination (Figure 2(F, f″)). Hypertrophic, phagocytic Kupffer cells have been identified in the sinusoids. A good histologic arrangement existed for the portal triads of structures, blood vessels, and stroma. In addition to reactive bile duct proliferation (biliary hyperplasia), which is a characteristic of ductular response, liver sections from AZOF-treated rats (Figure 2(A, a″)) demonstrated marked vascular dilatation with occasional interstitial hemorrhages, cellular disorganization, apoptosis of a variable number of cells and focal degenerative and necrotic changes. Hepatoportal biliary proliferation, round cells (lymphocytes and plasma cells) infiltration, and scattered hepatocellular degenerative and apoptotic changes were recorded in AZON-treated rats (Figure 2(B, b″)). Mild interstitial round cells infiltration with apparently normal hepato-portal and hepatocellular histo-morphological structures were seen in ZnNPs (Figure 2(C, c″)) and AZOF + ZnNPs treated rats (Figure 2(D, d″)). The same changes were recorded in AZON + ZnNPs treated rats with a moderate reactive portal round cell infiltration (Figure 2(E, e″)).

**Figure 3.**
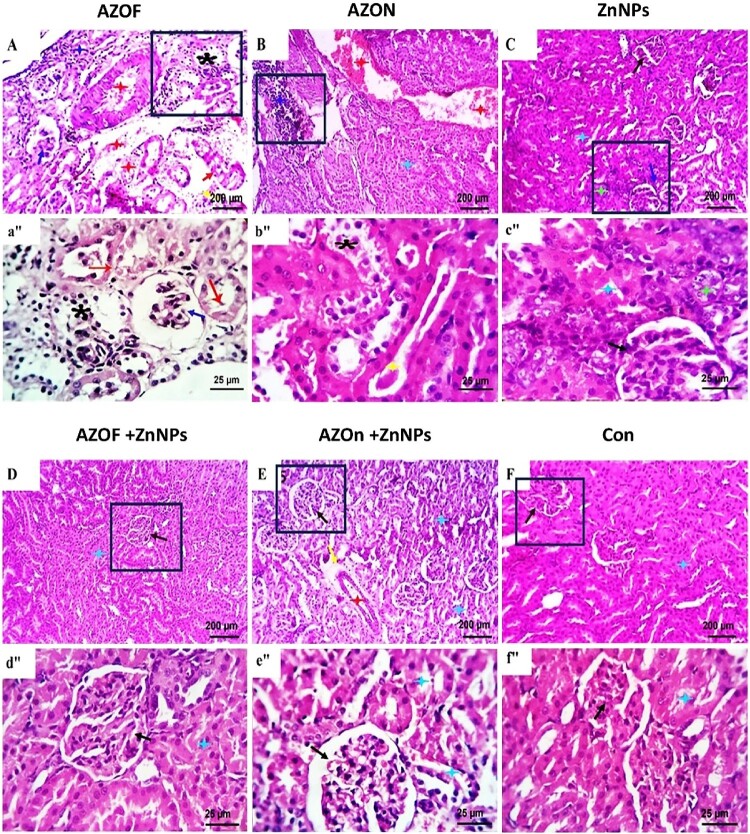
Photomicrographs from kidneys of different experimental groups showing (F, f″): normal histological structures of nephron units (black arrows and light blue asterisks). AZOF-treated rats (A, a″) showed marked vascular dilatation (red asterisks), edema (yellow arrow), and inflammatory cellular infiltration, mainly lymphocytes (dark blue asterisk). Tubular epithelial hydropic degeneration (black asterisk) and focal coagulative necrosis (red arrows) are also seen. Skunked hypocellular glomeruli (dark blue arrows), vascular dilatation (red asterisks), perivascular lymphocytic aggregations (dark blue asterisks) beside epithelial hydropic degeneration (black asterisk), intratubular hyaline casts formation, and tubular dilatation (yellow asterisk) are seen in AZON-treated rats (B, b″). ZnNPs (C, c″) treated rats show normal glomerular (black arrow) and tubular structures (light blue asterisk) with focal mitotically reactive tubular epithelia (nuclear basophilia) (green asterisk). AZOF + ZnNPs-treated rats (D, d″) show preserved glomerular and tubular structures (back arrow and light blue asterisk); meanwhile, mild perivascular edema (red asterisk, yellow arrow) was the only observable change in AZON + ZnNPs-treated rats (E, e″). (H&E stain). Scale bar in (A–F): 200 µm, (a″–f″): 25 µm.

The renal sections of control rats were shown in Figure 3(F, f″), showing the typical histological features of nephron structures, collecting ducts, renal papilla, pelvis, and stromal cells. AZOF-treated rats (Figure 3(A, a″)) revealed marked vascular dilatation, perivascular hemorrhages, edema, and inflammatory cellular infiltration, mainly lymphocytes. A moderate number of the glomeruli were skunked and appeared hypocellular. Along with the formation of intratubular hyaline casts and tubular dilatation, other conditions were seen, including tubular epithelial hydropic degeneration, focal coagulative necrosis, and scattered apoptosis. Rats treated with AZON also showed glomerular shrinkage, lobulation, vascular dilatation, perivascular lymphocytic aggregations, scattered tubular epithelial hydropic degeneration, apoptosis, and focal coagulative necrosis in addition to intratubular hyaline cast formation and tubular dilatation (Figure 3(B, b″)). Sections from ZnNP-treated rats (Figure 3(C, c″)) revealed normal glomerular and tubular structures with focal mitotically reactive tubular epithelia (nuclear basophilia). AZOF + ZnNPs-treated rats (Figure 3(D, d″)) showed micromorphological changes comparable to control-free rats with preserved glomerular and tubular structures. In contrast, mild perivascular edema was the only observable change in AZON + ZnNPs treated rats (Figure 3(E, e″)).

### 3.5 Immuno-histochemical findings

#### 3.5.1 Apoptotic marker (Caspase 3)

**Figure 4.**
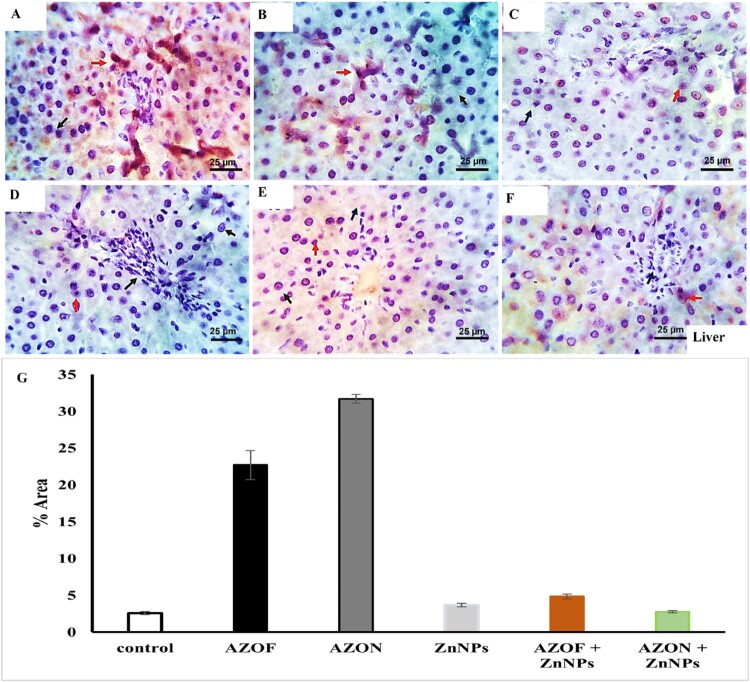
Photomicrographs from the liver of caspase 3 immune-stained tissue sections of control-free rats (F) and that of ZnNPs (C), AZOF + ZnNPs-treated rats(D), AZON + ZnNPs-treated rats (E) showing a very few numbers of positive weak brownish cytoplasmic stained cells (red arrows). Caspase 3 immune-stained tissue sections from the liver of AZOF-treated rats (A) and that of AZON-treated rats (B) showed moderate and mild to moderate numbers of positive brownish cytoplasmic stained cells, respectively (red arrows). Black arrows indicate negative cells. Scale bar: 25 µm. (G) demonstrating the estimated positive immune stained cells for caspase 3 expression in different experiment groups.

Caspase-3 immunohistochemistry labeling was utilized to evaluate the changes in liver tissue apoptosis. The number of positive weak brownish cytoplasmic stained cells was deficient in immune-stained tissue sections from livers of the control-free rats (Figure 4(F)), rats treated with ZnNPs (Figure 4(C)), AZOF + ZnNPs-treated rats (Figure 4(D)), and rats treated with AZON + ZnNPs (Figure 4(E)). Investigated immune-stained tissue sections from the liver of AZOF-treated rats (Figure 4(A)) and that of AZON-treated rats (Figure 4(B)) showed moderate and mild to moderate numbers of positive brownish cytoplasmic stained cells, respectively.

**Figure 5.**
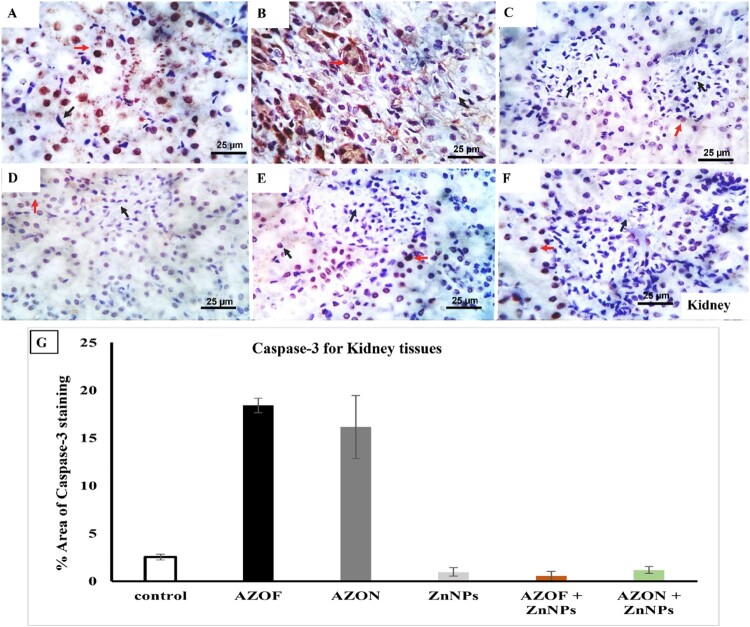
Photomicrographs from kidneys of caspase 3 immune-stained tissue sections of control-free rats (F) and that of ZnNPs (C), AZOF + ZnNPs-treated rats (D), AZON + ZnNPs-treated rats (E) showing a very few numbers of positive weak brownish cytoplasmic stained cells (red arrows). Caspase 3 immune-stained tissue sections from kidneys of AZOF-treated rats (A) and that of AZON-treated rats (B) showing mild to moderate and moderate numbers of positive brownish cytoplasmic stained cells respectively (red arrows). Black arrows indicate negative cells. (G) demonstrating the estimated positive immune stained cells for caspase 3 expression in different experiment groups. Scale bar: 25 µm.

However, immuno-stained tissue sections from the kidneys of untreated rats (Figure 5(F)), rats treated with ZnNPs (Figure 5(C)), rats treated with AZOF + ZnNPs (Figure 5(D)), and rats treated with AZON + ZnNPs (Figure 5(E)) revealed a very few numbers of positive, weakly stained cells with a brownish cytoplasm. The number of positive brownish cytoplasmic stained cells was mild to moderate in the kidneys of the rats treated with AZOF (Figure 5(A)) and moderate in the kidneys of the rats treated with AZON (Figure 5(B)).

#### 3.5.2 Morphometric analytic data

The degree of cell apoptosis in the liver tissue of several experimental groups was assessed by morphometric analysis of caspase-3 immune-stained sections. After exposure to AZOF and AZON (Figure S2), the immunological expression of Caspase-3 was increased in liver sections. In contrast, Caspase-3 was decreased in rats treated with ZnNPs, AZOF + ZnNPs, AZON + ZnNPs, and untreated animals. These results were supported statistically (Figure 4(G)). The percentage area of positive immune stained cells for caspase-3 expression was significantly higher in liver of AZOF-treated rats and AZON-treated rats (22.71 ± 1.956 and 31.71 ± 0.589 respectively) than in control animals (2.587 ± 0.205). The percent area of Caspase-3 staining was remarkably decreased in treatments of ZnNPs-treated rats, AZOF + ZnNPs-treated rats, and AZON + ZnNPs-treated rats (3.7 ± 0.224, 4.867 ± 0.332, and 2.772 ± 0.171, respectively) (Figure 4(G)). Furthermore, the morphometric analysis of caspase-3 immune-stained section from kidneys of different experimental groups revealed estimated positive cells as 22.7, 31.71, 3.7, 4.86, 2.77, and 2.58 in AZOF-treated rats (Figure S3), AZON-treated rats, ZnNPs-treated rats, AZOF + ZnNPs-treated rats, AZON + ZnNPs-treated rats, and untreated animals, respectively. In comparison to the other treatments (0.96 ± 0.442, 0.53 ± 0.514, 1.17 ± 0.375, and 2.53 ± 0.295) of ZnNPs-treated rats, AZOF + ZnNPs-treated rats, AZON + ZnNPs-treated rats, and untreated animals (Figure 5(G)), rats treated with AZOF or AZON had a higher percentage of immune-stained cells in their kidney tissues that were positive for caspase-3 expression (18.42 ± 0.761 and 16.17 ± 3.297), respectively.

### 3.6. Clustering heatmap and principal component analysis (PCA)

**Figure 6.**
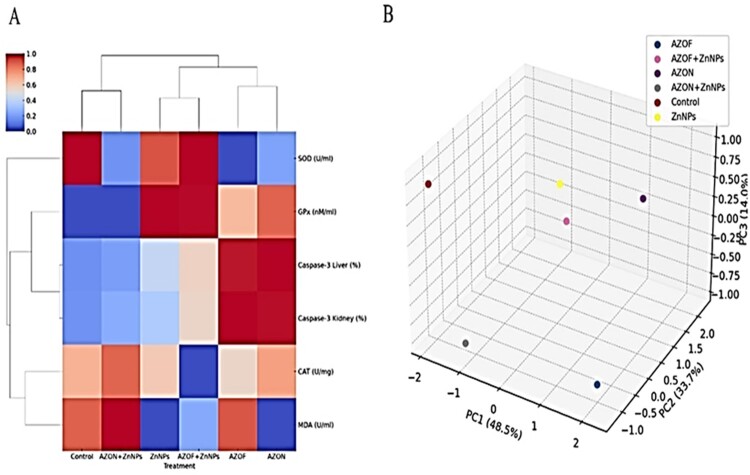
The heatmap clusters the treatments and the measured variables based on their standardized values. The treatments are clustered along the vertical axis and the variables along the horizontal axis. Red indicates higher values, and blue indicates lower values relative to the mean. (B) 3D PCA score plot for the experimental design. Each point represents a different treatment, plotted in the space of the first three principal components that capture the most variance in the data.

Figure 6 shows the clustering heatmap, which suggests that the AZOF treatment notably increases Caspase-3 activity, which may imply higher levels of apoptosis or cellular stress, especially in the liver. Conversely, antioxidant enzymes such as SOD, GPx, and CAT are generally lower with AZOF, hinting at potential oxidative stress. Combining AZOF with ZnNPs mitigates some of these effects, evidenced by lower Caspase-3 activity and a reduction in MDA levels, a marker of oxidative damage. ZnNPs alone also show a protective trend, with impact closer to the control. Meanwhile, the AZON treatment exhibits intermediate effects, which are not as protective as ZnNPs but less stressful than AZOF. Overall, ZnNPs might be protective against the stress induced by AZOF. The 3D PCA score plot with principal components explaining a cumulative 96.2% variance reveals a distinct clustering of treatments, indicative of their differential impacts on the measured parameters. Specifically, the separation along PC1 suggests that Control and ZnNP treatments have the most unique effects. At the same time, the proximity of AZOF to AZOF + ZnNPs and AZON to AZON + ZnNPs indicates that adding ZnNPs does not drastically alter the biochemical impact of AZOF or AZON treatments. These relationships suggest nuanced interactions between the treatments and the biological system under study, with ZnNPs potentially modulating the response to AZOF and AZON.

## 4. Discussion

The foremost global issue is the indiscriminate and reckless use of agrochemicals, which threatens human health and causes several environmental issues. Numerous observations predict that nanoscience and nanotechnology will enable the development of highly advanced agricultural fields that use nanoproducts [48], resulting in precise control and less pesticide use. Nano-pesticides are the most effective alternative to traditional pesticides [49]. Additionally, several studies indicated that nanoparticles can mitigate environmental and human dangers [6, 22, 50]. However, the nanomaterials' cytotoxicity and genotoxicity are also approaching [50]. Few studies were recorded on the mammalian toxicity of Az, and there is no information available about the toxicity of its nano-form on mammals. Therefore, it is essential to evaluate the effects of AZ and AZ-nano-form on the hepatic and nephron systems and identify ZnNPs as a protective biosynthesized agent to offset AZ-induced toxicity.

The decrease in PDI value that occurs when the concentration of aniseed oil is increased may be due to the thickener's higher effective concentration in the aqueous phase. PDI values below 0.25 indicate a narrow particle size distribution and excellent physical stability of the nanoemulsion since less Ostwald ripening occurs [52]. On the other hand, when the continuous phase's viscosity increases, the mobility of the oil droplet decreases, delaying the beginning of instability phenomena and producing oil droplets with more uniform particle sizes [35].

Significant levels of stability are often indicated by zeta potential values larger than +25 mV or lower than −25 mV [53]. Due to the applied field and Brownian motion, Z-average diameters of less than 20 nm suggest that the solution is very mobile [54]. Also, conductivity values of 5 mS/cm or more support the existence of highly conductive ions in the nano-emulsion, which may result in electrode polarization and deterioration [55].

SOD had a significant role in catalyzing the conversion of superoxide ions to H_2_O_2_. CAT was primarily responsible for the transition from H_2_O_2_ to H_2_O [56, 57]. Consequently, SOD and CAT were regarded as the most critical antioxidant enzymes for defending organisms against oxidative damage [58, 59].

It has been documented that different cells and tissues exhibit oxidative stress when Zn levels are inadequate; subsequently, Zn supplementation may help avoid oxidative damage. Zn protects cells from oxidative stress by supporting the biosynthesis of GPx, triggering the production of metallothioneins, and inhibiting NADPH oxidase in its role as a cofactor of the antioxidant enzyme SOD [19].

The present study shows that rats exposed to AZOF and AZON individually have significantly less SOD activity, which may lead to higher O_2_ concentrations. The accumulation of O_2_ has been demonstrated to decrease CAT activity. CAT is the primary hydrogen peroxide scavenger, and SOD activity is correlated with CAT activity. This information is consistent with that of El-Hak, Al-Eisa [9], who claimed that AZ exposure reduced SOD and CAT activities. The reduction in SOD and CAT activity may be attributed to the overproduction of reactive oxygen species (ROS) enzyme, indicating the antioxidant defense system failed to eliminate ROS influx [60]

Because AST, ALT, and ALP enzymes are biomarkers of liver damage, they have been employed as indicators for hepatotoxicity and health hazards. The current study evaluated the liver's health using serum ALT and AST indicators. Results revealed that AZOF-treated animals exhibit a remarkable increase in AST and a considerable decrease in ALT, which aligns with findings from Ziada, Abdulrhman and Nahas [10]. Rats receiving AZOF and ZnNPs concurrently had slightly elevated AST and ALT levels. These results are consistent with several studies [21, 24, 25] investigating how ZnNPs can modulate the alteration in ALT and AST enzymes of rats exposed to pesticides.

Interestingly, neither the ALT nor AST levels were markedly changed in rats treated with ZnNPs alone or the nano-form of AZ (AZON). It is noteworthy that nano-azoxystrobin was able to reduce the SOD and CAT activities of the fungus *Colletotrichum higginsianum* Sacc [17]. Unlike other groups, ALT and AST activity was suppressed when ZnNPs were administered along with AZON. This effect may be attributed to the ability of nanoparticles to adsorb and carry other substances, such as pesticides, which can improve the delivery of formulations [61]. Moreover, nanoparticles can serve as adjuvants to deliver chemical or biological pesticides, which can enhance the efficiency of those pesticides [62, 63]. Our results indicated that AZOF-intoxicated rats supplemented with ZnNPs rescued cells from oxidation damage by diminishing tissue MDA and stimulating the levels of GPx and SOD, which had been depleted by AZOF treatment. These findings were also reported by Bashandy, Alaamer [36], who demonstrated that ZnNPs can shield cells from oxidative injury caused by thioacetamide exposure by increasing the levels of the antioxidant enzymes CAT and SOD and lowering the levels of MDA. Anan, Zidan [64] and Aioub, Abdelnour [24] illustrated that ZnNPs administration in rats mitigated the toxic effect of cyclophosphamide and abamectin by activating CAT, SOD, and GPx levels and near-normalize the MDA levels.

According to histological liver and kidney sections from the rats treated with AZ, the current investigation showed that changes in hepato-renal biomarkers were a sign of early renal hepatocyte injury. According to current findings, rats exposed to AZOF or AZON revealed several histopathological signs in their liver and kidney. Inflammation-induced by AZ has been detected as significant hemorrhage and vasodilatation in liver and kidney tissues. In addition, degenerative and necrotic changes, cell apoptosis, hypocellular glomeruli, and intratubular hyaline cast formation were all seen. These results are supported by the findings of El-Hak, Al-Eisa [9] and Ziada, Abdulrhman and Nahas [10], who reported inflammatory cell infiltration, proliferation of fibroblasts, and degeneration of tubular epithelial cells in liver and kidney tissue.

By eliminating free radicals, ZnNPs had a cytoprotective impact on contaminants. They reversed the detrimental histopathological alterations in the analyzed hepato-renal tissues of the AZOF + ZnNPs and AZON + ZnNPs treated rats [65, 66]. These findings are verified by others [24, 67], proving the potency of ZnNPs as an antioxidant. This ZnNP property is validated by Lokman, Ashraf [68], who demonstrated that ZnONPs were associated with their capacity to scavenge free radicals, as well as to suppress apoptosis and inflammation caused by chronic aluminum uptake. Additionally, ZnNPs can mitigate fibrosis by reducing hepatic lipid peroxidation [36] and exhibit anti-inflammatory properties by inhibiting TNF-α from chlorpyrifos toxicity [66]. The wide range of impacts caused by metal nanoparticles is not surprising. For instance, titanium nanoparticles decreased the bioavailability of AZ in zebrafish larvae, which in turn lessened its effects on heart shape and dysfunction in the presence of high concentration of AZ.

Apoptosis, defined by morphological and genetic changes in the cell, is a type of programmed cell death. However, this process occurs under healthy conditions and chemicals-induced cell death in pathological circumstances [69]. The current study proves that AZOF and AZON induce apoptosis in liver and kidney cells via increasing immunological Caspase-3 expression. In contrast, Caspase-3 immuno-expression was considerably suppressed by ZnNP therapy, whether administered alone or in combination with AZOF or AZON. Hence, ZnNPs prevent apoptosis in the liver and kidney.

Similarly, AZ-induced apoptosis in neurons is associated with the upregulation of caspase 3 expression [70]. Furthermore, AZ can trigger apoptosis in zebrafish larvae by inducing caspase 3/caspase 9 activity in the mitochondrial pathway [71]. Huber and Hardy [72] reported that Zn inhibits caspase-3 activity, acting as an anti-apoptotic agent. Similar results were obtained by Mohammed, Safwat [25], who illustrated that ZnNPs could ameliorate hepatocyte apoptosis induced by atrazine through Bax and caspase-3 expressions. Moreover, Bax and caspase-3 markers were downregulated in kidney tissue after administering ZnNPs, as shown by Awadalla, Hussein [73]. Because of the potent antioxidant and antiapoptotic properties of ZnNPs, it can be used to reduce the harmful effects of Azoxystrobin fungicide and its nanoform on the liver and kidney.

Table 2 provides a comprehensive overview of the observed changes across all treatment groups compared to the control. The conventional azoxystrobin formulation (AZOF) induced marked increases in liver enzyme activity (ALT, AST), elevated oxidative stress (↑MDA), reduced antioxidant enzyme levels (↓SOD, ↓CAT), and severe histopathological and apoptotic alterations in liver and kidney tissues. The nanoformulation (AZON) also triggered oxidative and apoptotic responses but to a lesser extent. In contrast, zinc nanoparticles (ZnNPs) exhibited minimal changes and no observable toxicity. Co-administration of ZnNPs with either AZOF or AZON significantly mitigated most of the adverse effects, particularly by reducing caspase-3 expression and restoring antioxidant enzyme levels. These findings highlight the protective role of ZnNPs against azoxystrobin-induced hepato-renal toxicity and oxidative stress, reinforcing their potential as a safe detoxifying agent in pesticide exposure models.

**Table 2.** Summary of changes across treatments relative to control.

Parameter/Treatment	AZOF	AZON	ZnNPs	AZOF + ZnNPs	AZON + ZnNPs
ALT	↑	↑	→	↑	↓
AST	↑	→	→	↓	↓
SOD	↓	↓	↑	↑	↓
CAT	↓	↑	↑	↑	↑
GPx	↑	↑	→	→	↑
MDA	↑	↓	↓	↓	↓
Histology - Liver	Severe damage	Moderate damage	Normal	Mild damage	Minimal damage
Histology - Kidney	Severe damage	Moderate damage	Normal	Mild damage	Minimal damage
Caspase-3 - Liver	↑↑	↑	→	↓	↓
Caspase-3 - Kidney	↑	↑	→	↓	↓

↑, significant increase; ↓, significant decrease; →, no significant change; ↑↑, strong increase. Interpretation of ‘damage’ is based on qualitative histopathological findings.

## 5. Conclusion

Based on the findings, it is anticipated that the Azoxystrobin formulation and its nanoformulation can induce hepato-renal dysfunction by interfering with enzymes and triggering apoptosis by activating the apoptotic biomarker caspase-3 associated with oxidative stress. ZnNPs pretreatment, however, has shown significant antioxidant, anti-inflammatory, and anti-apoptotic properties, suggesting that ZnNP could be recommended to mitigate hepato-renal toxicity induced by both azoxystrobin and nano-azoxystrobin. Nevertheless, further studies are necessary to determine the effects of various doses of ZnNPs across different exposure scenarios. Additionally, research should address potential toxicity in other sensitive organs, particularly the lungs, which are highly vulnerable to oxidative stress, and assess the neuro- and testicular toxicity of ZnNPs in male albino rats. Understanding the mechanisms of ZnNPs' protective effects against Azoxystrobin is also essential to inform safe and effective application.

## Supplementary Material

Supplementary.docx

## Data Availability

The original contributions reported in the study are included in the article and further inquiries can be sent to the corresponding author.

## References

[1] Hassan AA, Salah KBH, Fahmy EM, et al. Olive leaf extract attenuates chlorpyrifos-induced neuro-and reproductive toxicity in male albino rats. Life. 2022;12(10):1500. 36294935 10.3390/life12101500 PMC9605092

[2] Morton V, Staub T. A short history of fungicides. APSnet Features. 2008;308:1–12.

[3] Kasanen R, Awan HUM, Zarsav A, *et al*. Forest tree disease control and management. In Asiegbu FO, Kovalchuk A, editors. Forest microbiology. Amsterdam: Elsevier. 2022; p. 425–462.

[4] Bartett DW, et al. Understanding the strobilurin fungicides. Pestic Outlook. 2001;12(4):143–148.

[5] Wang X, Li X, Wang Y, et al. A comprehensive review of strobilurin fungicide toxicity in aquatic species: emphasis on mode of action from the zebrafish model. Environ Pollut. 2021;275:116671. doi:10.1016/j.envpol.2021.116671 33582629

[6] Cycoń M, Piotrowska-Seget Z, Kozdrój J. Responses of indigenous microorganisms to a fungicidal mixture of mancozeb and dimethomorph added to sandy soils. Int Biodeterior Biodegrad. 2010;64(4):316–323. doi:10.1016/j.ibiod.2010.03.006

[7] Gao A-H, Fu Y-Y, Zhang K-Z, et al. Azoxystrobin, a mitochondrial complex III Qo site inhibitor, exerts beneficial metabolic effects in vivo and in vitro. Biochim Biophys Acta (BBA) - Gen Subj. 2014;1840(7):2212–2221. doi:10.1016/j.bbagen.2014.04.002 24726979

[8] Fernández-Ortuño D, Torés JA, de Vicente A, et al. Mechanisms of resistance to QoI fungicides in phytopathogenic fungi. Int Microbiol. 2008;11(1):1. 18683626

[9] El-Hak HNG, Al-Eisa RA, Ryad L, et al. Mechanisms and histopathological impacts of acetamiprid and azoxystrobin in male rats. Environ Sci Pollut Res. 2022;29(28):43114–43125. doi:10.1007/s11356-021-18331-3 PMC9148279 35091933

[10] Ziada RM, Abdulrhman SM, Nahas A. Hepato-nephro-toxicity induced by premium fungicide and protective effect of sesame oil. Egypt J Hosp Med. 2020;81(7):2445–2450. doi:10.21608/ejhm.2020.133961

[11] Cao F, Zhu L, Li H, et al. Reproductive toxicity of azoxystrobin to adult zebrafish (Danio rerio). Environ Pollut. 2016;219:1109–1121. doi:10.1016/j.envpol.2016.09.015 27616647

[12] Han Y, Liu T, Wang J, et al. Genotoxicity and oxidative stress induced by the fungicide azoxystrobin in zebrafish (Danio rerio) livers. Pestic Biochem Physiol. 2016;133:13–19. doi:10.1016/j.pestbp.2016.03.011 27742356

[13] Kah M, Hofmann T. Nanopesticide research: current trends and future priorities. Environ Int. 2014;63:224–235. doi:10.1016/j.envint.2013.11.015 24333990

[14] He C, Yin L, Tang C, et al. Size-dependent absorption mechanism of polymeric nanoparticles for oral delivery of protein drugs. Biomaterials. 2012;33(33):8569–8578. doi:10.1016/j.biomaterials.2012.07.063 22906606

[15] Kumar Singh S, Vaidya Y, Gulati M, et al. Nanosuspension: principles, perspectives and practices. Curr Drug Delivery. 2016;13(8):1222–1246. doi:10.2174/1567201813666160101120452 26721266

[16] Walker GW, Kookana RS, Smith NE, et al. Ecological risk assessment of nano-enabled pesticides: a perspective on problem formulation. J Agric Food Chem. 2017;66(26):6480–6486. doi:10.1021/acs.jafc.7b02373 28812885 PMC6152609

[17] Yao J, Cui B, Zhao X, et al. Preparation, characterization, and evaluation of azoxystrobin nanosuspension produced by wet media milling. Appl Nanosci. 2018;8:297–307. doi:10.1007/s13204-018-0745-5

[18] Powell SR. The antioxidant properties of zinc. J Nutr. 2000;130(5):1447S–1454S. doi:10.1093/jn/130.5.1447S 10801958

[19] Ruz M, Carrasco F, Rojas P, et al. Zinc as a potential coadjuvant in therapy for type 2 diabetes. Food Nutr Bull. 2013;34(2):215–221. doi:10.1177/156482651303400210 23964394

[20] Rasmussen JW, Martinez E, Louka P, et al. Zinc oxide nanoparticles for selective destruction of tumor cells and potential for drug delivery applications. Expert Opin Drug Deliv. 2010;7(9):1063–1077. doi:10.1517/17425247.2010.502560 20716019 PMC2924765

[21] Mansour SA, Abbassy MA, Shaldam HA. Zinc ameliorate oxidative stress and hormonal disturbance induced by methomyl, abamectin, and their mixture in male rats. Toxics. 2017;5(4):37. doi:10.3390/toxics5040037 29207507 PMC5750565

[22] Nie H, Pan M, Chen J, et al. Titanium dioxide nanoparticles decreases bioconcentration of azoxystrobin in zebrafish larvae leading to the alleviation of cardiotoxicity. Chemosphere. 2022;307:135977. doi:10.1016/j.chemosphere.2022.135977 35948095

[23] Rajput VD, Singh A, Minkina T, et al. Nano-enabled products: challenges and opportunities for sustainable agriculture. Plants. 2021;10(12):2727. doi:10.3390/plants10122727 34961197 PMC8707238

[24] Aioub AA, Abdelnour SA., Shukry M, et al. Ameliorating effect of the biological Zinc nanoparticles in abamectin induced hepato-renal injury in a rat model: implication of oxidative stress, biochemical markers and COX-2 signaling pathways. Front Pharmacol. 2022;13:947303. doi:10.3389/fphar.2022.947303 36172185 PMC9510891

[25] Mohammed ET, Safwat GM, Bahnasawy EA, et al. Zinc oxide nanoparticles and vitamin C ameliorate atrazine-induced hepatic apoptosis in rat via CYP450s/ROS pathway and immunomodulation. Biol Trace Elem Res. 2023;11:1–15. 10.1007/s12011-023-03587-2 PMC10509061 36790584

[26] Dyshlyuk L, Babich O, Ivanova S, et al. Antimicrobial potential of ZnO, TiO_2_ and SiO_2_ nanoparticles in protecting building materials from biodegradation. Int Biodeterior Biodegrad. 2020;146:104821. doi:10.1016/j.ibiod.2019.104821

[27] Singh M, Hemant KSY, Ram M, et al. Microencapsulation: A promising technique for controlled drug delivery. Res Pharm Sci. 2010;5(2):65. 21589795 PMC3093624

[28] Reis CP, Neufeld RJ, Ribeir, AJ, et al. Nanoencapsulation I. Methods for preparation of drug-loaded polymeric nanoparticles. Nanomed Nanotechnol Biol Med. 2006;2(1):8–21. doi:10.1016/j.nano.2005.12.003 17292111

[29] Nair R, Varghese SH, Nair BG, et al. Nanoparticulate material delivery to plants. Plant Sci. 2010;179(3):154–163. doi:10.1016/j.plantsci.2010.04.012

[30] Khot LR, Sankaran S, Maja JM, et al. Applications of nanomaterials in agricultural production and crop protection: a review. Crop Prot. 2012;35:64–70. doi:10.1016/j.cropro.2012.01.007

[31] Kong I. Polymers with nano-encapsulated functional polymers. design and applications of nanostructured polymer blends and nanocomposite systems. Boston: William Andrew Publishing; 2016. p. 125–154.

[32] Yang S, Yuan W, Jin T. Formulating protein therapeutics into particulate forms. Expert Opin Drug Deliv. 2009;6(10):1123–1133. 10.1517/17425240903156374 19663629

[33] Saravanakkumar D, Sivaranjani S, Umamaheswari M, et al. Green synthesis of ZnO nanoparticles using *Trachyspermum ammi* seed extract for antibacterial investigation. Der Pharma Chem. 2016;8(7):173–180.

[34] Meva FEA, Segnou ML, Okalla Ebongue C, et al. Spectroscopic synthetic optimizations monitoring of silver nanoparticles formation from Megaphrynium macrostachyum leaf extract. Rev Bras Farmacogn. 2016;26:640–646. doi:10.1016/j.bjp.2016.06.002

[35] Arancibia C, Navarro-Lisboa R, Zúñiga RN, et al. Application of CMC as thickener on nanoemulsions based on olive oil: physical properties and stability. Int J Polym Sci. 2016; 2016:1–10.

[36] Bashandy SA, Alaamer A, Moussa SAA, et al. Role of zinc oxide nanoparticles in alleviating hepatic fibrosis and nephrotoxicity induced by thioacetamide in rats. Can J Physiol Pharmacol. 2018;96(4):337–344. doi:10.1139/cjpp-2017-0247 28813612

[37] Nishikimi M, Rao NA, Yagi K. The occurrence of superoxide anion in the reaction of reduced phenazine methosulfate and molecular oxygen. Biochem Biophys Res Commun. 1972;46(2):849–854. doi:10.1016/S0006-291X(72)80218-3 4400444

[38] Paglia DE, Valentine WN. Studies on the quantitative and qualitative characterization of erythrocyte glutathione peroxidase. J Lab Clin Med. 1967;70(1):158–169. 6066618

[39] Ohkawa H, Ohishi N, Yagi K. Assay for lipid peroxides in animal tissues by thiobarbituric acid reaction. Anal Biochem. 1979;95(2):351–358. doi:10.1016/0003-2697(79)90738-3 36810

[40] Lowry OH, Rosebrough NJ., Farr AL, et al. Protein measurement with the Folin phenol reagent. J Biol Chem. 1951;193(1):265–275. doi:10.1016/S0021-9258(19)52451-6 14907713

[41] Aebi, H. [13] Catalase in vitro. In: Methods in enzymology. Amsterdam, The Netherlands: Elsevier; 1984; Vol. 105, p. 121–126. 10.1016/s0076-6879(84)05016-3 6727660

[42] Reitman S, Frankel S. A colorimetric method for the determination of serum glutamic oxalacetic and glutamic pyruvic transaminases. Am J Clin Pathol. 1957;28(1):56–63. doi:10.1093/ajcp/28.1.56 13458125

[43] Suvarna KS, Layton C, Bancroft JD. Bancroft’s theory and practice of histological techniques. London: Elsevier Health Sciences; 2018.

[44] Eissa S, Shoman S. Tumor markers. (No Title); 1998.

[45] Cattoretti G, Becker MHG, Key G, et al. Monoclonal antibodies against recombinant parts of the Ki-67 antigen (MIB 1 and MIB 3) detect proliferating cells in microwave-processed formalin-fixed paraffin sections. J Pathol. 1992;168(4):357–363. doi:10.1002/path.1711680404 1484317

[46] Hsu S-M, Raine L, Fanger H. Use of avidin-biotin-peroxidase complex (ABC) in immunoperoxidase techniques: a comparison between ABC and unlabeled antibody (PAP) procedures. J Histochem Cytochem. 1981;29(4):577–580. doi:10.1177/29.4.6166661 6166661

[47] Hashish H, Kamal R. Effect of curcumin on the expression of Caspase-3 and Bcl-2 in the spleen of diabetic rats. J Exp Clin Anat. 2015;14:18–23. doi:10.4103/1596-2393.158923

[48] Terzi E, Kartal SN, Yılgör N, et al. Role of various nano-particles in prevention of fungal decay, mold growth and termite attack in wood, and their effect on weathering properties and water repellency. Int Biodeterior Biodegrad. 2016;107:77–87. doi:10.1016/j.ibiod.2015.11.010

[49] Padmavathi P, Vasundhara N, Kovvuri S, et al. Synthesis and characterization of nano-acetamiprid—new plant safeguard nanomaterial. Am J Anal Chem. 2020;11(5):197–204. doi:10.4236/ajac.2020.115015

[50] Refat MS, Hamza RZ, Adam AMA, et al. Antioxidant, antigenotoxic, and hepatic ameliorative effects of quercetin/zinc complex on cadmium-induced hepatotoxicity and alterations in hepatic tissue structure. Coatings. 2021;11(5):501. doi:10.3390/coatings11050501

[51] Chaud M, Souto EB., Zielinska A, et al. Nanopesticides in agriculture: benefits and challenge in agricultural productivity, toxicological risks to human health and environment. Toxics. 2021;9(6):131. doi:10.3390/toxics9060131 34199739 PMC8230079

[52] Hoeller S, Sperger A, Valenta C. Lecithin based nanoemulsions: a comparative study of the influence of non-ionic surfactants and the cationic phytosphingosine on physicochemical behaviour and skin permeation. Int J Pharm. 2009;370(1-2):181–186. doi:10.1016/j.ijpharm.2008.11.014 19073240

[53] Mahobia S, Bajpai J, Bajpai AK. An in-vitro investigation of swelling controlled delivery of insulin from egg albumin nanocarriers. Iran J Pharm Res. 2016;15(4):695. 28243266 PMC5316248

[54] Berne BJ, Pecora R. Dynamic light scattering: with applications to chemistry, biology, and physics. New York, NY, USA: Courier Corporation; 2000.

[55] Patakangas J. Investigation of electrolyte materials and measurement techniques for nanocomposite fuel cells. 2014.

[56] Shigeoka S, Ishikawa T, Tamoi M, et al. Regulation and function of ascorbate peroxidase isoenzymes. J Exp Bot. 2002;53(372):1305–1319. doi:10.1093/jexbot/53.372.1305 11997377

[57] Mittler R. Oxidative stress, antioxidants and stress tolerance. Trends Plant Sci. 2002;7(9):405–410. doi:10.1016/S1360-1385(02)02312-9 12234732

[58] Asada K. The water-water cycle in chloroplasts: scavenging of active oxygens and dissipation of excess photons. Annu Rev Plant Physiol Plant Mol Biol. 1999;50(1):601–639. doi:10.1146/annurev.arplant.50.1.601 15012221

[59] Gill SS, Tuteja N. Reactive oxygen species and antioxidant machinery in abiotic stress tolerance in crop plants. Plant Physiol Biochem. 2010;48(12):909–930. doi:10.1016/j.plaphy.2010.08.016 20870416

[60] Ali D, Ibrahim KE, Hussain SA, et al. Role of ROS generation in acute genotoxicity of azoxystrobin fungicide on freshwater snail *Lymnaea luteola* L. Environ Sci Pollut Res. 2021;28:5566–5574. doi:10.1007/s11356-020-10895-w 32974827

[61] Jong D, H W, Borm PJ. Drug delivery and nanoparticles: applications and hazards. Int J Nanomed. 2008;3(2):133–149. doi:10.2147/IJN.S596 PMC2527668 18686775

[62] Mali SC, Raj S, Trivedi R. Nanotechnology a novel approach to enhance crop productivity. Biochem Biophys Rep. 2020;24:100821. doi:10.1016/j.bbrep.2020.100821 33015378 PMC7522746

[63] Yan S, Ren BY, Shen J. Nanoparticle-mediated double-stranded RNA delivery system: A promising approach for sustainable pest management. Insect Sci. 2021;28(1):21–34. doi:10.1111/1744-7917.12822 32478473

[64] Anan HH, Zidan RA, Abd EL-Baset SA, et al. Ameliorative effect of zinc oxide nanoparticles on cyclophosphamide induced testicular injury in adult rat. Tissue Cell. 2018;54:80–93. doi:10.1016/j.tice.2018.08.006 30309514

[65] Atef HA, Mansour MK, Ibrahim EM, et al. Efficacy of zinc oxide nanoparticles and curcumin in amelioration the toxic effects in aflatoxicated rabbits. Int J Curr Microbiol Appl Sci. 2016;5(12):795–818. doi:10.20546/ijcmas.2016.512.090

[66] Prasad AS. Zinc is an antioxidant and anti-inflammatory agent: its role in human health. Front Nutr. 2014;1:14. doi:10.3389/fnut.2014.00014 25988117 PMC4429650

[67] Navaei-Nigjeh M, Gholami M, Fakhri-Bafghi MS, et al. Molecular and biochemical evidences for beneficial effects of zinc oxide nanoparticles in modulation of chlorpyrifos toxicity in human lymphocytes. Iran J Pharm Res. 2018;17(3):927. 30127816 PMC6094429

[68] Lokman M, Ashraf E, Kassab RB, et al. Aluminum chloride–induced reproductive toxicity in rats: the protective role of zinc oxide nanoparticles. Biol Trace Elem Res. 2022;200(9):4035–4044. doi:10.1007/s12011-021-03010-8 34741695

[69] Elmore S. Apoptosis: a review of programmed cell death. Toxicol Pathol. 2007;35(4):495–516. doi:10.1080/01926230701320337 17562483 PMC2117903

[70] Kang J, Bishayee K, Huh S-O. Azoxystrobin impairs neuronal migration and induces ROS dependent apoptosis in cortical neurons. Int J Mol Sci. 2021;22(22):12495. doi:10.3390/ijms222212495 34830376 PMC8622671

[71] Jiang J, Wu S, Lv L, et al. Mitochondrial dysfunction, apoptosis and transcriptomic alterations induced by four strobilurins in zebrafish (Danio rerio) early life stages. Environ Pollut. 2019;253:722–730. doi:10.1016/j.envpol.2019.07.081 31344535

[72] Huber KL, Hardy JA. Mechanism of zinc-mediated inhibition of caspase-9. Protein Sci. 2012;21(7):1056–1065. doi:10.1002/pro.2090 22573662 PMC3403442

[73] Awadalla A, Hussein AM, El-Far YM, et al. Effect of zinc oxide nanoparticles and ferulic acid on renal ischemia/reperfusion injury: possible underlying mechanisms. Biomed Pharmacother. 2021;140:111686. doi:10.1016/j.biopha.2021.111686 34015581

[74] Söğüt İ, Senat A, Oglakci Ilhan A, et al. Evaluation of thiol/disulfide homeostasis and other oxidative stress markers in patients undergoing hemodialysis. Turk J Nephrol. 2021;30(1):17–24. doi:10.5152/turkjnephrol.2021.4396

