# Supplementary material for: Zinc nanoparticles mitigate azoxystrobin and its nanoencapsulation-induced hepatic and renal toxicity in rats
Source: Redox Rep. 2025 Apr 20;30(1):2491318. doi: 10.1080/13510002.2025.2491318 (PMC12010655; doi:10.1080/13510002.2025.2491318)
Supplement: Supplementary.docx [file YRER_A_2491318_SM2651.docx]

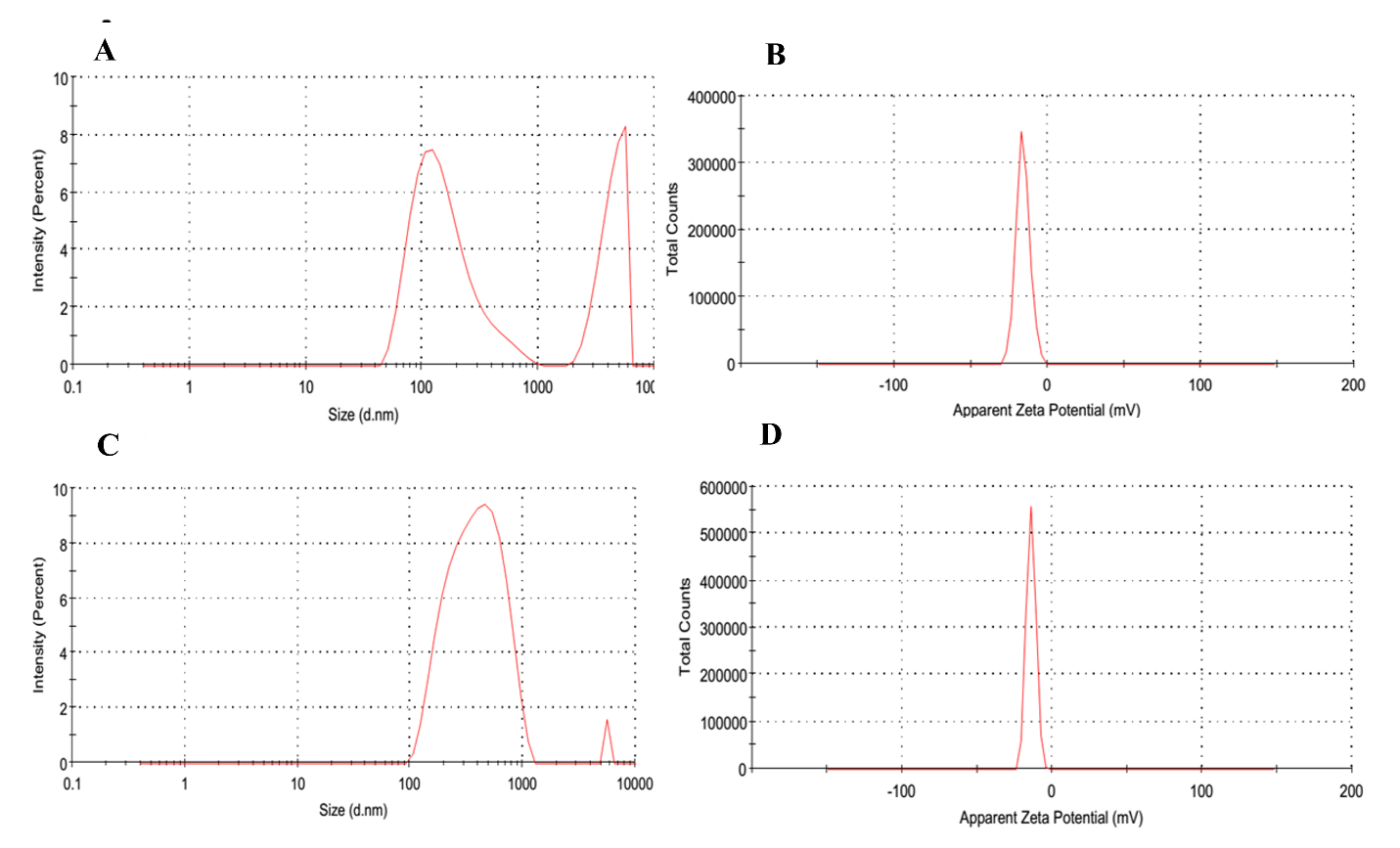
**Figure S1:** Size distribution by intensity (A) and zeta potential (B) of ZnNPs sample. Size distribution by intensity (C) and zeta potential (D). Nano- azoxystrobin sample


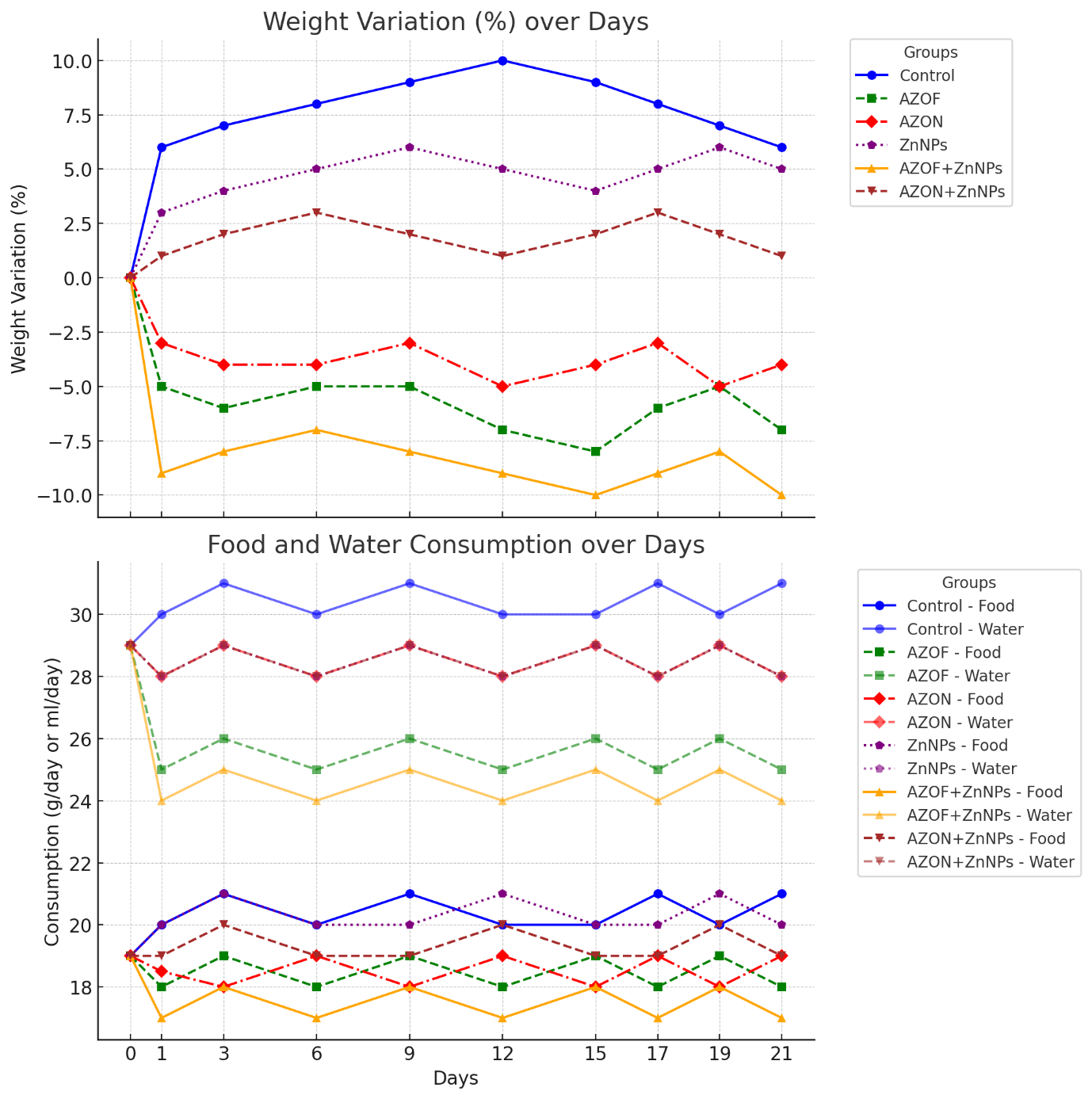
Figure S2 illustrates trends in weight variation and consumption patterns across different treatment groups over time. Control animals show a consistent increase in body weight, likely due to stable conditions. In contrast, treatments such as AZOF and AZOF+ZnNPs demonstrate significant reductions in weight variation, possibly due to reduced metabolic efficiency or adverse effects on growth. Interestingly, groups treated with ZnNPs and AZON+ZnNPs exhibit minimal or positive weight changes, suggesting potential protective or compensatory effects of ZnNPs. Food and water consumption patterns align with weight variation trends, where control and ZnNP-treated animals maintain stable intake. At the same time, AZOF-treated groups show decreased food and water consumption, potentially reflecting stress or toxicity. These findings indicate that AZOF may negatively impact metabolic health, while ZnNPs may have protective properties when combined with other treatments, warranting further investigation into the mechanisms underlying these effects.

Primary monoclonal and polyclonal antibodies for caspase-3 (Cat. No. PAI-29157, Thermo Fisher Scientific Co., USA), respectively were added after dilution by PBS (2 μg/ml and 1:1000, respectively) and incubated for 30 min. The slides were washed three times for 3 min each with PBS. Biotinylated polyvalent secondary antibody (Cat. No. 32230, Thermo Scientific Co., UK) was applied to tissue sections and co-incubated for 30 min.
